# Basic Psychological Needs and Sports Satisfaction Among Brazilian Athletes and Coaches: The Mediating Role of the Dyadic Relationship

**DOI:** 10.3389/fpsyg.2019.02543

**Published:** 2019-11-12

**Authors:** Andressa Ribeiro Contreira, José Roberto Andrade do Nascimento Junior, Nayara Malheiros Caruzzo, Luciane Cristina Arantes da Costa, Patrícia Aparecida Gaion, Sandro Victor Alves Melo, Lenamar Fiorese

**Affiliations:** ^1^Grupo de Estudos de Psicologia do Esporte e Desempenho Humano, Physical Education Department, Universidade Estadual de Maringá, Maringá, Brazil; ^2^Grupo de Estudos em Psicologia do Esporte e do Exercício, Physical Education Department, Universidade Federal do Vale do São Francisco, Petrolina, Brazil; ^3^Duke Global Health Institute, Duke University, Durham, NC, United States; ^4^Health and Sports Science Center, Universidade Federal do Acre, Rio Branco, Brazil

**Keywords:** interpersonal relationships, satisfaction, motivation, sport, psychological need

## Abstract

Even though sport satisfaction has proved an important element for youngsters to keep practicing sports, little is known on the sport satisfaction of coaches. Moreover, the coach-athlete relationship is acknowledged as a key element for sport success, but whether its importance is the same for coaches and athletes is yet to be investigated. Our study analyzed the mediating role of the coach-athlete relationship in associating the satisfaction of basic psychological needs and sport satisfaction of Brazilian coaches and athletes. 364 coaches and athletes participated in the study representing 182 dyads from different sports according to the following instruments: Basic Needs Satisfaction Sport Scale (BNSSS), Coach-Athlete Relationship Questionnaire, athlete and coach versions (CART-Q), and the Athletic Satisfaction Questionnaire (ASQ). Data analysis followed a Structural Equation Modeling (SEM) with a significance level of *p* < 0.05, generating results in which the mediating model for coaches was not adequately fit, while the direct model, without mediation, was adequately fit and explained 48% of sport satisfaction variance. For athletes, the mediating model has shown adequate fit and explained 81% of the sport satisfaction variance, leading us to conclude that the quality of the coach-athlete relationship can be considered a determining factor for the satisfaction of young Brazilian athletes’ basic psychological needs as well as sport satisfaction, but proved not as relevant to their coaches.

## Introduction

Studies in the high-performance sport context have intensively focused on factors to contribute to the well-being of athletes considering that the environment favors tense situations and emotional alterations (Balaguer et al., 2012). In this context, the Theory of Self-determination (TAD) points out to the satisfaction of basic psychological needs (autonomy, competence, and relationships) as key factors to achieve well-being by encompassing universally essential elements to the integrity of human development (Deci and Ryan, 2012). Recent studies have indicated that when feeling independent to control emotions and truly connected to a social environment through the support of their coaches, athletes are more likely to reach self-determined motivation and consequently feel satisfied with their sports (Deci et al., 2013; Reynoulds and Mcdonough, 2015; Gurrola et al., 2016).

Such evidence is supported by the micro theory of Basic Psychological Needs (Deci and Ryan, 2012), which states that social environments conducted by significant persons (such as teachers, coaches, parents) favor the satisfaction of athletes’ basic needs for providing them with psychological experiences that positively affect their motivation and performance (Balaguer et al., 2012; Deci et al., 2013; González et al., 2015). In contrast, poorly adapted environments can frustrate the need of youngsters and consequently lower their participation in sports and sense of personal fulfilment, in addition to generate both emotional and physical fatigue (Bartholomew et al., 2011; Gurrola et al., 2016).

Recent studies have pointed out that athletes’ satisfaction with team structural and procedural aspects, such as group environment and sport experiences, is associated with the optimization of both cognitive and emotional performance (Riemer and Chelladurai, 1998; Jowett and Poczwardowski, 2007; Lee et al., 2017; Ntomali et al., 2017; Rocchi and Pelletier, 2018). Additionally, athletes’ satisfaction favors the development of group cohesion (García-Calvo et al., 2014; Kim and Cruz, 2016), intrinsic motivation (Mageau and Vallerand, 2003; Cranmer and Sollitto, 2015), establishment of effective communication (Sullivan and Gee, 2007; Kao and Tsai, 2016), and well-being (Kim and Cruz, 2016; Jowett et al., 2017).

In this perspective, we highlight the relevance of sport satisfaction to the well-being and performance of athletes, which has been explained according to variables such as leadership styles (Kao and Tsai, 2016; Kim and Cruz, 2016; Ntomali et al., 2017), social support (Cranmer and Sollitto, 2015), and motivational environment (Bekiari and Syrmpas, 2015). The behavior of sport leaders has proved a determinant role in athletes’ satisfaction (Kao and Tsai, 2016) and social support is important when transmitting information from coaches for contributing to the emotional support of athletes (Cranmer and Sollitto, 2015). In addition, a motivational environment acts as catalyst for athletes’ motivation regarding their engagement in sports and consequently their satisfaction with sport experiences (Bekiari and Syrmpas, 2015).

In an effort to understand how motivation and sport satisfaction relate to positive experiences and the optimization of athletes’ performance (Vallerand, 2000; Kao and Tsai, 2016; Lee et al., 2017), our study bases on the TAD to investigate natural or intrinsic human tendencies to behave in an efficient, healthy manner. The TAD remains one of the most commonly used theoretical approaches and approaches the reasons leading an individual to initiate, remain or quit their activities (Ryan and Deci, 2017). The micro theory of Basic Psychological Needs predicts universally essential elements for human development, motivation, integrity, and general well-being emerging upon the satisfaction of basic psychological needs regarding autonomy, competence, and personal relationships (Ryan and Deci, 2017).

Specifically, autonomy corresponds to the individual’s ability to feel in control of their actions and decisions. Competence refers to the need of an individual to feel efficient enough to reach their desired results. Personal relationships are based on an innate capacity of individuals to perceive themselves truly connected to a social environment (Deci and Ryan, 2012; Reynoulds and Mcdonough, 2015). Researches indicate that athletes who see themselves as able to manage their actions within the sport context feel physically and psychologically able to performe their functions and feel accepted in their teams, in addition to being more likely to feel intrinsically motivated, thus facilitating the perception of sport satisfaction (Deci et al., 2013; Bekiari and Syrmpas, 2015; Reynoulds and Mcdonough, 2015; Gurrola et al., 2016; Monteiro et al., 2018; Vieira et al., 2018).

However, despite the important impact that motivation can have on athletes’ satisfaction (Mageau and Vallerand, 2003; Bekiari and Syrmpas, 2015; Lee et al., 2017), most studies have focused on athletes (Cranmer and Sollitto, 2015; Kao and Tsai, 2016; Kim and Cruz, 2016; Ntomali et al., 2017) rather than the perspective of sport coaches (Lorimer, 2009; Lorimer and Jowett, 2009; Kim and Cruz, 2016). It is known that even more important than the isolate perception of athletes or coaches, their combined views, characterized as dyads, can provide more efficient and thorough information on sport experiences (Lorimer and Jowett, 2009; Jowett, 2017).

The quality of the coach-athlete relationship is considered a central axis in the sport context defined as a combination of inter-relations involving thoughts, feelings, and behaviors of coaches and athletes (Jowett and Poczwardowski, 2007; Jowett, 2017). Such relationship has been studied from the integrated model 3+1Cs (Jowett and Poczwardowski, 2007) incorporating affective (closeness), cognitive (commitment), and behavior (complementarity) components of the dyad, in addition to the degree to which athletes’ and coaches’ perceptions interconnect (co-orientation) (Rhind and Jowett, 2012).

Studies have reported that close dyadic relationships based on respect, affection, and commitment benefit the development of athlete excellence contributing to the personal growth of both athletes and coaches (Cheuczuk et al., 2016; Davis et al., 2018; Avci et al., 2018). In contrast, dyads characterized by distance and absence of commitment imply interpersonal conflicts, exhaustion, dissatisfaction, and lack of interest inside and outside the sport context (Jowett and Lavalee, 2007; Antonini et al., 2011; Li et al., 2015; Jowett and Shanmugam, 2016; Davis et al., 2018).

Our study considers the importance of motivational aspects for athletes’ satisfaction and the impact of social psychology in the sport context (Jowett and Poczwardowski, 2007) and seeks to improve the scientific knowledge discussing the implications in the dyadic relationships to promote positive psychological aspects for coaches and young Brazilian athletes taking into account affective, cognitive, and behavior aspects, representing the gap investigated in our study *per se*. The fostering of harmonious sport environments generates potential literature evidence regarding athletes’ development (Jowett, 2017; Ryan and Deci, 2017); however, it is yet to be clarified whether such development is also applicable to coaches (Lorimer, 2009; Lorimer and Jowett, 2009; Kao and Tsai, 2016).

Thus, the analysis of positive psychological variables in the perspective of the main social actors in the sport context (coach and athlete) enables a larger view on the personal and interpersonal factors that focus on their well-being and performance beyond the perspective of isolated influences from coaches’ behavior. Considering these remarks, our goal was to investigate the mediating impact of the coach-athlete relationship on the association between basic psychological needs and athletes’ satisfaction of coaches and young Brazilian athletes.

### Hypothesis

Our first hypothesis involves a positive association between sport motivation, characterized by the satisfaction of basic psychological needs, and sport satisfaction for both coaches and athletes considering that autonomous and competent individuals who also feel connected to their peers are more likely to feel satisfied (González et al., 2015). Our second hypothesis considers that coach-athlete relationship will increase the impact of motivation over sport satisfaction since positive coach-athlete relationships may improve these individuals’ physical and psychological well-being providing them with better performance and satisfaction with the sport (Jowett and Shanmugam, 2016).

## Materials and Methods

### Participants

Aiming at representing the dyadic relationships, we calculated the sample size by considering the limiting number of coaches participating in the competition, which was performed for finite samples with a 95-confidence level and a five-percent confidence interval (Richardson et al., 2012). We included a total of 540 coaches to insure the participation of at least 159 dyads (*n* = 159 coaches; *n* = 159 athletes). Inclusion criteria as follows: (1) having qualified for the national stage of the main youth sporting event in Brazil, the Youth School Games; (2) a coach-athlete relationship of over 3 months (Hampson and Jowett, 2014; Sagar and Jowett, 2015; Nicholls and Perry, 2016). We selected the athletes intentionally after sampling the coaches. Based on the inclusion criteria for the coaches – team and individual sport types – coaches were requested to indicate an athlete who met criterion 3 (a 3-month old relationship) to constitute the dyad.

A total of 189 dyads participated in the study (378 subjects), however, seven pairs were excluded for having answered the questionnaires incorrectly, which generated a final total of 364 individuals representing 182 dyads. The average age in the samples was 40.47 ± 9.7 years old for coaches and 16.24 ± 0.81 years old for athletes. The individuals represented all five regions of Brazil and 13 different sports: volleyball (*n* = 30; 16.5%), judo (*n* = 30; 16.5%), basketball (*n* = 29; 15.9%), handball (*n* = 23; 12.6%), track and field (*n* = 22; 12.1%), futsal (*n* = 12; 6.6%), swimming (*n* = 12; 6.6%), Olympic wrestling (*n* = 7; 3.8%), table tennis (*n* = 5; 2.7%), chess (*n* = 5; 2.7%), cycling (*n* = 3; 1.6%), rhythmic gymnastics (*n* = 3; 1.6%), and beach volleyball (*n* = 1; 0.5%).

### Measures

We used the Brazilian version of the Basic Needs Satisfaction in Sport Scale (Nascimento Junior et al., 2018), originally developed by Ng et al. (2011), to investigate the satisfaction of basic psychological needs of coaches and athletes. Differently from the original scale (which has five dimensions: competence, choice, internal perceived locus of causality, volition, and relatedness), the Brazilian scale is constituted of only one general dimension of autonomy. It was pointed out as a necessary change during the various phases of the instrument adaptation to the Portuguese language, such as at the content analysis phase and factor structure analysis (EFA and CFA). In the EFA, the authors tested different models in order to find the best fit since the content analysis revealed inconsistencies. Thus, the instrument is composed of 12 items distributed in three dimensions: competence (item 6 “I feel that I am good at my sport”), autonomy (item 1 “In my sport, I fell that I am pursuing my personal goals”) and relatedness (item 4 “There are people in my sport who care about me”), which is available for reader consultation (Nascimento Junior et al., 2018). Answers are given in a 7-point Likert scale ranging from 1– “Not true at all” to 7– “Very true.” Scale Confirmatory Factor Analysis (CFA) presented acceptable fit for athletes [*X*^2^(51) = 82.137; *p* = 0.004; *X*^2^/*df* = 1.611; CFI = 0.94; GFI = 0.93; TLI = 0.93; RMSEA = 0.06; *P*(rmsea < 0.05) = 0.268]. We submitted the scale to a process of content validation in order to adjust its items for coaches, which generated acceptable Content Validity Coefficient (CVC = 0.91). CFA analysis for coaches also revealed acceptable fit [*X*^2^(51) = 104.208; *p* = 0.001; *X*^2^/*df* = 2.043; CFI = 0.93; GFI = 0.92; TLI = 0.90; RMSEA = 0.07; *P*(rmsea < 0.05) = 0.023].

To measure coaches’ and athletes’ direct perspective of their social relationships, we adopted the Portuguese version of the Coach-Athlete Relationship Questionnaire (Vieira et al., 2015 athlete version – α > 0.70; Contreira et al., 2019 coach version – α = 0.86). The scale was originally developed by Jowett and Ntoumanis (2004) and comprises 11 items assessing the following three dimensions: closeness (item 3 “I like my coach/athlete”), commitment (item 2 “I am committed to my coach/athlete”), and complementarity (item 10 “In my training with my coach/athlete, I am willing to do my best”) Items answered in a seven-point Likert scale ranging from 1-“Strongly disagree” to 7-“Strongly agree.” CFA revealed instrument’s acceptable fit for athletes [*X*^2^(39) = 93.926; *p* = 0.001; *X*^2^/*df* = 2.408; CFI = 0.90; GFI = 0.92; TLI = 0.87; RMSEA = 0.08; *P*(rmsea < 0.05) = 0.04] and coaches [*X*^2^(37) = 76.284; *p* = 0.001; *X*^2^/*df* = 2.062; CFI = 0.90; GFI = 0.94; TLI = 0.88; RMSEA = 0.07; *P*(rmsea < 0.05) = 0.04].

We used the version of the (Borrego et al., 2010) Athlete Satisfaction Questionnaire (Riemer and Chelladurai, 1998) validated for the Portuguese language. This instrument assesses the level of athletes’ satisfaction with their sport experiences and is composed of 53 statements answered in a Likert scale ranging from 1-“Not at all satisfied” to 7-“Extremely satisfied,” encompassing a 15-level scale of satisfaction. In this study, we used a shorter 11-item version that assesses only the following three dimensions: training and instruction (item 7 “The training that I receive/give from/to my coach/athlete regarding technique and tactics of my position”), individual performance (item 1 “The level on which my performance goals were reached during the season”), and personal treatment (item 11 “How my coach/athlete supports me”), regarded as directly relevant for the coach-athlete relationship study (Jowett and Don Carolis, 2003; Jowett, 2008; Lorimer, 2009; Lorimer and Jowett, 2009). This same short version of the questionnaire had been used in a previous research involving coaches and athletes (Jowett, 2008; Lorimer, 2009; Jowett and Nezlek, 2011). The scale CFA showed acceptable fit for athletes [*X*^2^(41) = 69.852; *p* = 0.003; *X*^2^/*df* = 1.704; CFI = 0.95; GFI = 0.94; TLI = 0.93; RMSEA = 0.06; *P*(rmsea < 0.05) = 0.198]. With the help of a specialists committee, a version of the instrument was adapted for coaches and generated a satisfactory content validity (CVC > 0.80). Coach-version CFA also revealed acceptable fit indices [*X*^2^(32) = 98.184; *p* = 0.001; *X*^2^/df = 3.068; CFI = 0.92; GFI = 0.90; TLI = 0.90; RMSEA = 0.09; *P*(rmsea < 0.05) = 0.001].

### Procedure

This study was approved by the Ethics Committee of a Brazilian university under statement number 1.324.411/2015. All participants and legal representatives had read and signed an Informed Consent Term. Data gathering occurred during the Youth School Games, regarded as the most important competition for athletes at this age group in the country. We collected the data at the locations where the competitions were being held as well as in individuals’ accommodations according to their availability. The individuals answered the questions in group, but filling the questionnaire individually, averaging 20 min per person. Data collection with athletes and coaches occurred throughout the competition held in November 2015.

### Data Analysis

#### Preliminary Analysis

We performed an exploratory data analysis with descriptive statistics as mean (x), standard deviation (sd). We compared the BPN satisfaction, athletics satisfaction and CAR between athletes and coaches through an independent student *t*-test (*p* < 0.05). All analyses were performed using Amos 22.0.

#### Main Analysis

Our main goal was to verify whether CAR mediated the relationship between BPN satisfaction (independent variable) and athlete’s satisfaction (dependent variable) using a Structural Equation Modeling (SEM) on software Amos 22.0, following the two-step model building approach recommend by Anderson and Gerbing (1988). The first step involves testing the measurement model through a Confirmatory Factor Analysis (CFA), while in the second step the hypothesized structural model is tested.

The internal consistency of the measurement model (Step 1) was assessed by composite reliability (CR) (Hair et al., 2005), while average variance extracted (AVE) were estimated to assess convergent validity (Fornell and Larcker, 1981). A CR equal or higher than 0.7 and an AVE equal or higher than 0.5 are considered reliable and valid constructs (Fornell and Larcker, 1981). Discriminant validity was established whenever AVE for each construct exceeded the squared correlations between the construct in question and any other construct (Fornell and Larcker, 1981).

Before the main analysis, we verified the data for normality, missing values, and outliers for all study variables following the procedure outlined by Tabachnick and Fidell (2013). Examination of skewness and kurtosis for all variables indicated univariate normality based on the cut-off values of skewness < 3.0 and kurtosis < 10.0 (Kline, 2016). Analysis of Mardia’s multivariate coefficient (Athletes = 38.62; Coaches = 52.35) indicated that the data distribution derived from multivariate normality, which justified the use of the Bollen-Stine bootstrap procedure to obtain a corrected Chi-squared value (Athletes – *p* = 0.194; Coaches – *p* = 0.005) of the estimated coefficients for the Maximum Likelihood Estimator (Bollen and Stine, 1993). After excluding seven pairs (coaches and athletes), no missing data was identified. We verified the occurrence of outliers using the Square Mahalanobis distance (D^2^) since the absence of such cases is a prerequisite for this analysis.

We used several fit indices to assess the model fit according to Hu and Bentler (1999) recommendations: chi square (χ2), Normalized Chi-Square (χ2/df), Comparative Fit Index (CFI), Tucker-Lewis Index (TLI), Root Mean Square Error of Approximation (RMSEA), and its associated ninety-percent Confidence Interval (CI). CFI and TLI values close to or above 0.95, RMSEA values close to or below 0.08, and the lower end of 90% CI of the RMSEA containing the value of 0.05 represent an excellent fit to the data for the hypothesized model (Hu and Bentler, 1999). Furthermore, we used these indices for both Step 1 and Step 2. Fit quality for the structural model (Step 2) was also assessed through its factor loadings (FL) and items individual reliability (Marôco, 2010). Based on Kline’s recommendation (2016), the reference for path interpretation included small effect below 0.20; medium effect between 0.20 and 0.49; and large effect above 0.50 (*p* < 0.05).

#### Mediation Analysis

In order to test the theoretical model proposed for the study, the mediation effects were verified by the indirect effects (Williams and MacKinnon, 2008). Bias-corrected bootstrapped point estimates for the indirect effects of the independent variable on the dependent variable were estimated, considering 95% confidence intervals. Significant indirect effects were considered (at alfa = 0.05) if its 95% confidence intervals does not include zero. Bias corrected and accelerated intervals supported by a 1000 samples bootstrapping were used to make inferences. Bootstrapping procedures have been recommended Williams and MacKinnon (2008) as more efficient and powerful detecting indirect effects in smaller samples.

## Results

### Descriptive Results

The brazilian coach and athlete sociodemographic profile are shown in Table 1. Most of the coaches were male (82.4%), whereas most of athletes were female (51.6%). Over half of coach-athletes dyades (62.6%) had one to 5 years of relationship. The majority of coaches has more than 10 years in the field (74.2%), while most athlete showed 1–5 years experience (48.1%).

**TABLE 1:** Sociodemographic profile of Brazilian coaches and athletes (*n* = 182 dyads).

**Variables**	***F***	**(%)**
**Gender**		
**Coaches**		
Male	150	82.4
Female	32	17.6
**Athletes**		
Male	88	48.4
Female	94	51.6
**Type of Sport**		
Individual	87	47.8
Teams	95	52.2
**Coach-athlete dyads**		
<1 year	07	3.8
1–5 years	114	62.6
>5 years	51	28.0
**Years experience**		
**Coaches**		
<5 years	08	4.4
5 a 10 years	36	19.8
>10 years	135	74.2
**Athletes**		
<1 year	02	1.1
1 a 5 years	87	48.1
>5 years	84	46.4
**Region of Brazil**		
South	23	12.6
Southeast	25	13.7
West center	28	15.4
Northeast	62	34.1
North	43	23.6

*Source: the authors.*

Table 2 shows the descriptive data for the main variables in the study. Athletes and coaches had different perceptions only for closeness, with athletes (6.69 ± 0.56) perceiving higher levels than coaches (6.54 ± 0.84) (*p* = 0.03). Regarding the basic needs =, we found that coaches (6.17 ± 0.76) felt more competent than athletes (5.97 ± 0.80) (*p* < 0.01), while athletes presented higher autonomy (6.66 ± 0.55) and relatedness (6.30 ± 0.82) than coaches (6.52 ± 0.63 and 5.84 ± 0.95, respectively) (*p* < 0.01). Athletes have also shown higher levels of satisfaction with training and instruction (6.41 ± 0.77) and personal treatment (6.47 ± 0.63) than coaches (6.18 ± 0.74 and 6.31 ± 0.67, respectively) (*p* < 0.01).

**TABLE 2:** Coach-athlete relationship, basic psychological needs and sport satisfaction comparison between coaches and athletes (*n* = 182 dyads).

**Variables**	**Coaches *X* (SD)**	**Athletes *X* (SD)**	***P***
**CAR**			
Closeness	6.54 (0.84)	6.69 (0.56)	**0.03^∗^**
Commitment	6.24 (0.81)	6.16 (0.84)	0.54
Complementarity	6.54 (0.61)	6.52 (0.58)	0.08
**BPN**			
Competence	6.17 (0.76)	5.97 (0.80)	**0.01^∗^**
Autonomy	6.52 (0.63)	6.66 (0.55)	**0.01^∗^**
Relatedness	5.84 (0.95)	6.30 (0.82)	**0.01^∗^**
**ST**			
Training-instruction	6.18 (0.74)	6.41 (0.77)	**0.01^∗^**
Individual performance	5.88 (0.77)	5.81 (0.83)	0.58
Personal treatment	6.31 (0.67)	6.47 (0.63)	**0.01^∗^**

*Independent sample *t-*test (^∗^*p* < 0.05). CAR (Coach-athlete relationship); BPN (basic psychological needs); ST (sport satisfaction). Source: the authors. The values in bold correspond to the significance level of the comparisons made. These values showed statistically significant differences between the groups.*

Table 3 shows the correlations between basic needs satisfaction, coach-athlete relationship and sport satisfaction. Coach data are displayed in the upper triangle and correlation values for athletes in the lower triangle. Significant correlations were obtained for most variables for coaches, while athletes presented correlations among all variables (*p* < 0.05). The strongest correlation between different constructs for coaches occurred between BPN’s competence and ST’s training and instruction (*r* = 0.57) as well as between CAR complementarity and ST personal treatment (*r* = 0.52).

**TABLE 3:** Correlation matrix for study variables.

**Variables**	**CAR**			**BPN**			**Athletic satisfaction**		
			
	**1**	**2**	**3**	**4**	**5**	**6**	**7**	**8**	**9**
1. Closeness	–	**0.49^∗^**	**0.42^∗^**	**0.21^∗^**	0.12	**0.15^∗^**	**0.24^∗^**	**0.43^∗^**	**0.47^∗^**
2. Commitment	**0.43^∗^**	–	**0.53^∗^**	**0.30^∗^**	**0.37^∗^**	**0.22^∗^**	**0.43^∗^**	**0.40^∗^**	**0.53^∗^**
3. Complementarity	**0.55^∗^**	**0.55^∗^**	–	**0.43^∗^**	**0.41^∗^**	**0.34^∗^**	**0.40^∗^**	**0.36^∗^**	**0.35^∗^**
4. Competence	**0.19^∗^**	**0.27^∗^**	**0.30^∗^**	–	**0.52^∗^**	**0.43^∗^**	**0.57^∗^**	**0.31^∗^**	**0.33^∗^**
5. Autonomy	**0.27^∗^**	**0.34^∗^**	**0.33^∗^**	**0.40^∗^**	–	**0.32^∗^**	**0.42^∗^**	**0.39^∗^**	**0.46^∗^**
6. Relatedness	**0.23^∗^**	**0.32^∗^**	**0.29^∗^**	**0.17^∗^**	**0.25^∗^**	–	**0.28^∗^**	**0.17^∗^**	**0.31^∗^**
7. Training-instruction	**0.42^∗^**	**0.42^∗^**	**0.46^∗^**	**0.25^∗^**	**0.25^∗^**	**0.32^∗^**	–	**0.63^∗^**	**0.51^∗^**
8. Individual performance	**0.24^∗^**	**0.36^∗^**	**0.29^∗^**	**0.35^∗^**	**0.28^∗^**	**0.22^∗^**	**0.48^∗^**	–	0.52
9. Personal treatment	**0.45^∗^**	**0.50^∗^**	**0.52^∗^**	**0.36^∗^**	**0.30^∗^**	**0.25^∗^**	**0.55^∗^**	**0.43^∗^**	–

*^∗^Pearson correlations, significant values for *p* < 0.05. Upper triangle (Correlation coefficients for coaches) – Lower triangle (Correlation coefficients for athletes). CART (1. Closeness; 2.Commitment; 3. Complementarity); BPN (4. Competence; 5. Autonomy; 6. Relatedness); ST (7. Training-instruction; 8. Individual performance; 9. Personal treatment). CAR: coach-athlete relationship subscales; BPN: Basic Psychological needs subscale. Source: the authors. Bold values correspond to statistically significant correlations between variables.*

### Measurement Model (Step 1)

Initially, we tested a three-factor measurement model through CFA (SEM Step 1) by assessing the relationship of the items analyzed with their respective latent factors. Acceptable fit indices were obtained for both coaches [*X*^2^(24) = 55.17; *p* = 0.001; *X*^2^/*df* = 2.51; CFI = 0.94; GFI = 0.94; TLI = 0.90; NFI = 0.90; RMSEA = 0.08; *P*(rmsea < 0.05) = 0.014] and athletes [*X*^2^(24) = 47.35; *p* = 0.003; *X*^2^/*df* = 1.97; CFI = 0.94; GFI = 0.95; TLI = 0.91; NFI = 0.90; RMSEA = 0.07; *P*(rmsea < 0.05) = 0.103]. Moreover, local adjustment and the internal reliability of items also proved adequate, since all paths had significant FL > 0.50. In this sense, the latent model was confirmed and enabled for SEM Step 2. In order to assess the convergent validity, AVE was computed. The AVE values were as follows for athletes and coaches, respectively,: BPN = 0.45/0.53; CAR = 0.47/0.50; and ST = 0.53/0.51. Only two variables showed AVE lower the cut-off, however, these values were very close to 0.50. We observed that the BPN revealed to be discriminant to the others (AVE > SC) for both athletes and coaches. CAR and ST revealed to be discriminant to the others (AVE > SC) just for coaches, while, for athletes, CAR and ST showed higher SC (0.76) than their AVE. This result can be related to the association between these variables, which assess similar constructs (Jowett and Don Carolis, 2003; Jowett, 2008; Lorimer, 2009). The composite reliability values were as follows for athletes and coaches: BPN = 0.65/0.78; CAR = 0.71/0.74; and ST = 0.78/0.75.

### Structural Equation Modeling (Step 2)

#### Direct Effect

**FIGURE 1:**
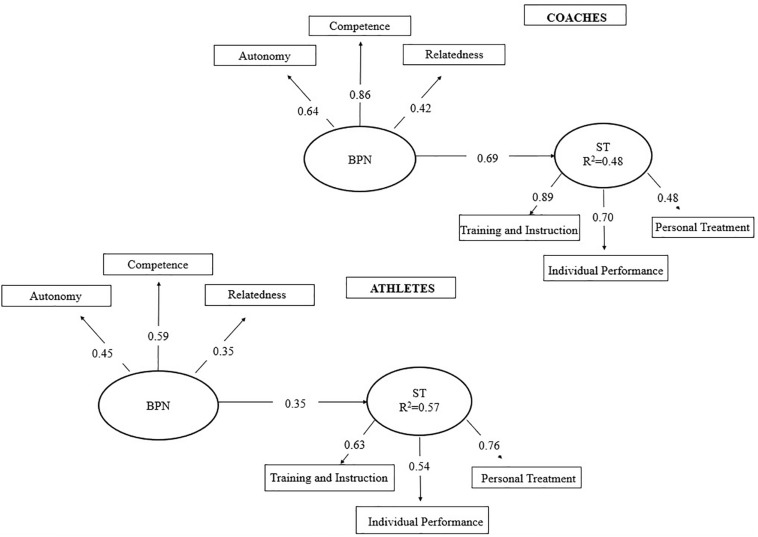
Structural equation model of BPN impact over coaches and athletes satisfaction (*n* = 182 dyads).

Firstly, we tested a model with direct paths between BPN and ST (Figure 1), which had adequate fit (Table 4) with BPN explaining 48% and 57% of ST variance for coaches and athletes, respectively.

**TABLE 4:** Fit indices comparison with and without mediation for coaches and athletes’ structural equation model (*n* = 182).

**Models**	**B/Sχ^2^**	**χ^2^/*df***	**RMSEA [95% C.I.]**	**CFI**	**TLI**
**Coaches**					
No mediation	23.34	2.92	0.10 [0.09–0.12]	0.95	0.90
CAR mediation	90.17	3.76	0.12 [0.10–0.13]	0.87	0.81
**Athletes**					
No mediation	14.37	2.05	0.08 [0.06–0.08]	0.95	0.90
CAR mediation	34.97	1.52	0.05 [0.04–0.06]	0.97	0.95

*B/S*X*^2^ = Bollen-stine Chi-Square; *df* = Degrees of freedom; *X*^2^/*df* = Normalized chi-square; RMSEA = Root mean square error of approximation; TLI = Tucker-lewis index; CFI = Comparative fitness index. Source: the authors.*

#### Indirect Effect

**FIGURE 2:**
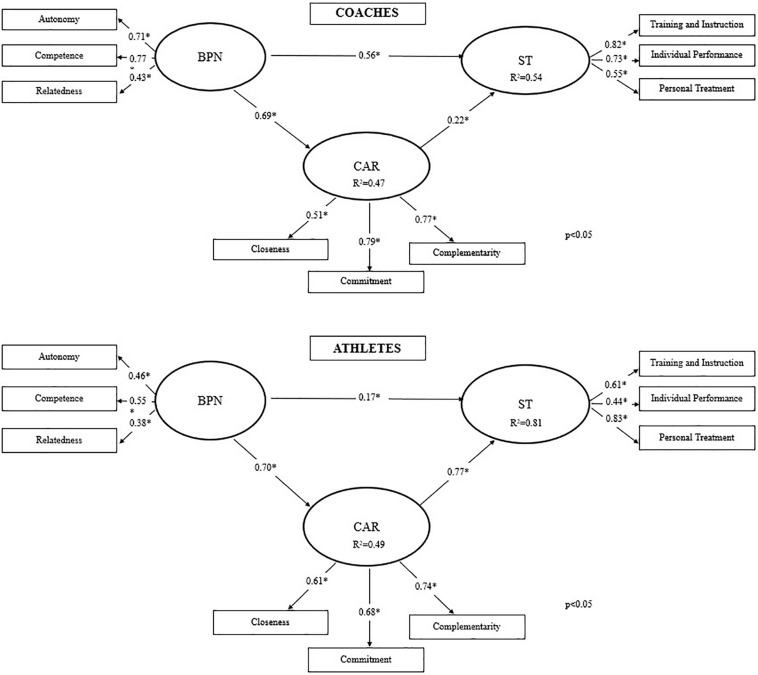
Structural equation modeling of the CAR mediating role over the BPN impact on coaches and athletes satisfaction (*n* = 182 dyads).

We tested a second model including CAR as a mediating variable over the BPN effect on satisfaction. The model for coaches did not show adequate fit (Table 4), still, model paths and shared variance indicated the CAR mediating role (Figure 2). Meanwhile, the model for athletes presented acceptable fit (Table 4) and evidenced a significant mild-strength mediating positive effect of CAR suggesting the importance of young athletes attributing it to the relationship with their coaches (Figure 2).

By analyzing Bootstrapped parameter estimates for coaches (Table 5), we found that their satisfaction (ST) had a 54% explained variance from BPN + CAR and CAR shared 47% of its variance with BPN. A strong positive effect occurred from BPN to ST (β = 0.56; *p* < 0.05) as well as a mild positive effect from CAR to ST (β = 0.22; *p* < 0.05). The impact of BPN over ST increased when mediated by the independent indirect CAR effect (β = 0.15) (total effect β = 0.71).

**TABLE 5:** Standardized direct and indirect effects for the structural model (M2) for coaches and athletes (*n* = 182 dyads).

**Parameter**			**Effect**	**95% CI**
**Coaches**				
**Direct effects**				
BPN	→	CAR	0.69	0.46–0.85
	→	ST	0.56	0.29–0.89
CAR	→	ST	0.22	0.08–0.48
Indirect effect of BPN via				
CAR	→	ST	0.15	0.04–0.29
Total effect			0.71	
**Athletes**				
**Direct effects**				
BPN	→	CAR	0.70	0.40–0.93
	→	ST	0.17	0.02–0.89
CAR	→	ST	0.77	0.33–1.46
**Indirect effect of BPN via**				
CAR	→	ST	0.54	0.31–0.76
Total effect			0.71	

*CI – Confidence interval; CAR (coach-athlete relationship); BPN (basic psychological needs); ST (sport satisfaction). ^∗^*p* < 0.05. Source: the authors.*

When analyzing the mediating Bootstrapped parameter estimates for athletes (Table 5), we observe that 81% of the variance in their satisfaction levels (ST) could be predicted by BPN + CAR and CAR was explained in 49% by the BPN. The direct path between BPN and ST had a weak positive effect (β = 0.17; *p* < 0.05) and a strong positive effect emerged from CAR to ST (β = 0.77; *p* < 0.05). CAR mediation revealed an independent and high indirect effect (β = 0.54) in the association (*p* < 0.05) (total effect β = 0.71).

## Discussion

Our aim was to examine the mediating role of coach-athlete relationship in the association between basic needs satisfaction and sport satisfaction of Brazilian coaches and athletes. Our Hypothesis 1 was confirmed: BPN had a positive impact over both coaches (48%) and athletes (57%) satisfaction with slightly more relevant impact for athletes. In contrast, our Hypothesis 2 was only partially supported since such mediating model presented acceptable fit exclusively for athletes. In this model, variance of athletes’ satisfaction was predicted in 81%. Although adequate fit indices were not reached, the model for coaches offered evidence regarding the role coach-athlete relationship in coach satisfaction.

### Effects of Basic Psychological Needs on Athletes’ Satisfaction

According to the literature, the impact of basic needs satisfaction over sport satisfaction revealed in the results confirm the predictions (Figure 1). According to the SDT, basic needs of autonomy, competence and relatedness are universal elements essential for human development, its integrity, and general well-being (Deci and Ryan, 2012) mainly due to their contribution to intrinsic motivation (Costa et al., 2016). A more autonomous motivation is known to be related to higher levels of satisfaction with performance, as found in a longitudinal study by Blecharz et al. (2015) involving professional Polish athletes. The study found a model explaining 57% of performance satisfaction variance and highlighted the significant indirect effect of intrinsic motivation mediating the association between self-efficacy and performance satisfaction for those athletes.

Another study approached collegiate athletes and found that those who receive support from had higher levels of satisfaction regarding their sports (Hoffmann and Loughead, 2016). Such evidence strengthen our findings by showing that when athletes feel engaged and connected to their social environment, they are more likely to present higher levels of satisfaction with their sport, which reveals how interpersonal relationships stablished in sport practices are intervening factors for athletic satisfaction.

Regarding the BPN impact over satisfaction of Brazilian coaches (Figure 1), our findings contribute to current knowledge on the subject with important advances considering that the field of sport psychology is yet to be further explored since most studies have focused only on athletes’ satisfaction (Lorimer, 2009; Kim and Cruz, 2016). Our results revealed that coaches’ BPN explained 48% of their sport satisfaction, similarly to the findings of Jowett (2008) when studying the impact of intrinsic and extrinsic motivation of coaches on their satisfaction. The author found positive effects from motivation to satisfaction upon the presence of self-determined intrinsic motivation. Rocchi and Pelletier (2018) found that coaches whose training contexts enable the satisfaction of their basic psychological needs tend to have an autonomous regulation of motivation as well as an interpersonal support attitude. In contrast, the frustration of such needs contributes to the controlled regulation of motivation and the absence of interpersonal support.

According to SDT, it is possible to state that coaches who have their basic needs met, especially regarding competence (Table 1), tend to reach greater satisfaction in their sport practice. Such positive evaluation of processes and outcomes regarding their experience can be explained by the SDT mini-theory of Cognitive Evaluation, which addresses the effects of social contexts on intrinsic motivation since contextual events have control aspects that influence competence perception (Lorimer, 2009; Ryan and Deci, 2017). Therefore, we understand that sport represents a social context in which a coach needs to feel capable of performing their tasks and responsibilities when coaching athletes and teams including effort and involvement in activities.

### The Mediating Role of the Coach-Athlete Relationship in Sport Motivation and Satisfaction

Our mediating model indicated that the study on coach-athlete relationship led to a higher degree of explained variance for sport satisfaction suggesting that it is important for athletes to establish a good relationship with their coaches in order to feel satisfied with the sport (Figure 2). Our results emphasize the relevance of a positive relationship between coaches and young athletes, especially regarding their perception of closeness (affective component), as well as the dimensions of training and instruction, and personal treatment (Table 1). A study by Cranmer and Sollitto (2015) supports our findings by demonstrating that social support from coaches predicted better satisfaction of college athletes with their sport experiences. Coaches are considered vital for the entire process of involvement in a sport for allowing athletes to reach their maximum potential (Kim and Cruz, 2016; Ramazanoğlu, 2018) and having the potential to shape athletes’ experiences in their sport context (Cranmer and Sollitto, 2015). Jowett et al. (2017) add that athletes who share a good-quality relationship with their coaches experience high levels of satisfaction of their basic needs, which establishes a positive prediction of self-determined motivation and well-being.

Considering our participants’ age group, our findings are supported by evidences in the literature that show how young athletes need experienced and well-qualified coaches who offer support and orientation to help them overcome challenges and adversities in their sport (Jackson et al., 2010). In this context, the strong mediation of CAR in athletic satisfaction agrees with SDT’s assumptions, which understands human behavior in interaction with the environment and how motivation will be influenced by athletes’ social contexts in which a positive participation of social agents, such as teachers, colleagues, family, and coaches, can make individuals more self-determined (Ryan and Deci, 2017).

Model 3+1C’s model corroborates such aspects and reveals that the relationship quality intensifies individual feelings of happiness and satisfaction upon affection reciprocity along with cognitive and behavioral aspects between coaches and athletes (Jowett and Shanmugam, 2016). Jowett and Nezlek (2011) add that close relationships of British coaches and athletes benefit satisfaction, especially for aspects of formation, training and instruction, and personal treatment, similarly to our findings.

Despite not having reached adequate fit, the mediating model for coaches presented significant paths between latent variables (Figure 2) indicating a potential of CAR to mediate the BPN association with coaches’ satisfaction, which is regarded as deriving from a positive affective condition based on the processes and outcomes of sport experiences (Lorimer, 2009; Kim and Cruz, 2016). Thus, we consider that even though important, CAR did not prove a determining factor for these Brazilian coaches to feel satisfied with their experience of coaching young athletes.

According to our findings, Lorimer (2009) found a model in which CAR predicted 32% of British coaches’ satisfaction, but with a major proportion of unexplained variance resulting from the multifaceted nature of CAR, which also varies according to specific goals and professionalism. According to Jowett (2008), complex extrinsic factors will dictate coaches’ satisfaction beyond their relationships with athletes alone, such as monetary rewards, contracts, need for recognition, competition level, organizational pressure, and the technical level of the team.

Other aspects intervening in the association between CAR and coaches’ satisfaction may result from the fact that some coaches base their sport experiences almost exclusively on training and instruction aspects, dedicating little time to social relationships (Lorimer, 2009). In this perspective, there is growing evidence in the literature highlighting the importance of developing positive interpersonal environments between coaches and athletes (Jowett and Poczwardowski, 2007; Jowett, 2017), which might reflect on harmonious relationships, satisfaction, psychological well-being, and improved performance for both sides. Therefore, we emphasized the need to foster social interactions between coaches and their young athletes.

## Conclusion and Limitations

Our study revealed that young athletes and coaches’ sport satisfaction is strongly influenced by the satisfaction of their basic psychological needs. For athletes, such relationship is much stronger when mediated by the coach-athlete relationship. Our findings contribute to coaches, sport psychologists, and other professionals involved with youth sports regarding their understanding on the importance of social relationships to motivate and provide young athletes with satisfaction; in addition, it is important to promote closeness, commitment along with training and instruction. It is important for coaches to include psychological aspects and social relationships in their training and competition atmospheres to overcome mere technical and tactical aspects. In this sense, it is important for athletes to feel motivated to persist in their sport that they also feel close to their coaches in an affective relationship involving respect and trust.

Even though our study provides empirical evidence on the importance of CAR as mediator in the relationships between the BPN and the satisfaction of coaches and young Brazilian athletes, it is important to point out some limitations. Among such limitations, we highlight language adequacy in the ASQ and BNSSS for coaches since these scales originally assessed sport satisfaction and BPN satisfaction in the perspective of athletes. Aiming at verifying the validity of such adequacy, the instruments were assessed by Ph.D. professors in Sport Psychology regarding the clarity of language and practical relevance to be later applied to a reduced sample of coaches before being used in the total sample of the research. Still, the CFA indicated acceptable indices of the factorial structure of the instruments adapted to coaches. We also highlight that despite such limitation, the adequacy (adaptation) of scales allows to advance in the scientific knowledge on satisfaction and the BPN for coaches, which becomes restrict due to the lack of instruments for this population. Finally, the transversal format of study enabled significant predictions on the relationships among the variables, but did not allow causal relations whereas a longitudinal would enable more robust inferences. In this context, further studies are suggested to use a longitudinal design to monitor the variables since the perceptions of the coaches and athletes modify throughout the sport season, or even during training and competitions. We also highlight that some variables, such as personality traits, competitive level, and significant achievements by coaches, may influence the CAR mediator effect. Furthermore, our study limits to verify associations focusing on a positive perspective and does not investigate the need frustration as a negative affective condition determined by a complex evaluation of the structures, processes, and results related to sports experience influencing the CAR. Considering the importance of analyzing these variables, we suggest that further studies should include such investigation.

## Data Availability Statement

The raw data supporting the conclusions of this manuscript will be made available by the authors, without undue reservation, to any qualified researcher.

## Ethics Statement

This study was carried out in accordance with the recommendations of Standing Committee on Ethics in Research with Humans, from the State University of Maringa with written informed consent from all subjects. All subjects gave written informed consent in accordance with the Declaration of Helsinki. The protocol was approved by the Standing Committee on Ethics in Research with Humans, under opinion 1.324.411/2015.

## Author Contributions

AC, LF, and JN provided the study with design and idea. AC, NC, PG, SM, and LC recruited and acquired data from participants. JN, AC, and PG analyzed and interpreted the data. AC, LF, NC, PG, LC, SM, and JN have contributed to the manuscript drafting. All authors have contributed to the study critical review and submission approval.

## Conflict of Interest

The authors declare that the research was conducted in the absence of any commercial or financial relationships that could be construed as a potential conflict of interest.
